# Influence of Drying Methods on Jackfruit Drying Behavior and Dried Products Physical Characteristics

**DOI:** 10.1155/2022/8432478

**Published:** 2022-09-05

**Authors:** Sophie Nansereko, John Muyonga, Yusuf B. Byaruhanga

**Affiliations:** Department of Food Technology and Nutrition, Makerere University, P.O. Box 7062, Kampala, Uganda

## Abstract

Drying processes including solar, oven, and refractance window were studied to determine their influence on the drying behavior of jackfruit slices and properties of resultant jackfruit powders. The loss of sample mass, converted to the ratio between the water content at time *t* and the initial water content (moisture ratio), was used as the experimental parameter for modelling drying processes. Fifteen thin layer drying models were fitted to the experimental data using nonlinear regression analysis. Based on the highest *R*^2^ and lowest SEE values, the models that best fit the observed data were Modified Henderson and Pabis, Verma et al., and Hii et al. for RWD, oven, and solar drying, respectively. The effective moisture diffusivity coefficients were 5.11 × 10^−9^, 3.28 × 10^−10^, and 2.55 × 10^−10^ for RWD, oven and, solar drying, respectively. The solubility of freeze-dried jackfruit powder (75.7%) was not significantly different from the refractance window dried powder (73.2%) and was higher than oven-dried jackfruit powder (66.1%). Oven-dried jackfruit powder had a lower rehydration ratio and porosity. Differences in rehydration ratio and porosity under different drying methods could be explained by the microstructure. Fractal dimension (FD) and lacunarity were applied to study the structure and irregularities of jackfruit dried with the different methods. FD was significantly (*P* < 0.05) affected by the drying method. FD ranged from 1.809 to 1.837, while lacunarity ranged between 0.258 and 0.404.

## 1. Introduction

Jackfruit (*Artocarpus heterophyllus Lam*) is an important fruit, extensively cultivated in tropical, subtropical, and temperate regions of the world [1]. The fruit and seeds are rich sources of minerals, vitamins, organic acids, and dietary fiber. Previous research has shown that jackfruit has anticarcinogenic, antimicrobial, antifungal, anti-inflammatory, wound healing, and hypoglycemic properties, all of which can be attributed to its diverse nutrient and biochemical profile [2]. Despite these benefits, the fruit is underutilized, is not listed as a commercial crop, and is rarely planted on a large scale due to its limited shelf life and lack of processing facilities in the regions where it is cultivated [3]. Since jackfruit is highly perishable, processing is needed to preserve the fruit and reduce postharvest losses. Minimal processing techniques, refrigeration, and dehydration or drying are among the useful processes used to preserve jackfruits [4],

Drying aims to remove as much water as possible to significantly reduce microbial spoilage and oxidation reactions [5]. Drying also minimizes packaging requirements and reduces product weight for ease of transportation [6]. Some drying methods applied to jackfruit include solar drying to make jackfruit leather [7], a combination of instant controlled pressure drop-assisted freeze-drying, instant controlled pressure drop assisted hot air drying, and freeze-drying to make jackfruit chips [8], hot air drying [9], osmotic dehydration [10, 11], drum drying [12, 13], osmo-convective drying [14], freeze-drying [15], and convection oven drying [16]. Refractance window drying, a novel drying technology, has recently been optimized for drying jackfruit with positive results [17]. Refractance window (RW) drying is a method that has been used for drying heat sensitive products such as fruit and vegetable purees, slices, and juices into powders, flakes, or sheets. RW comprises a thin film drying system with high heat and mass transfer rates that speed up the drying rate at a comparatively lower temperature. It uses circulating hot water at atmospheric pressure as a heating medium for the material to be dehydrated [18]. These drying technologies differ in drying speed, energy efficiency, product quality, dryer costs, and technological simplicity. The main technical challenge is to identify a relatively inexpensive drying technology that gives high-quality products, even from heat-sensitive materials, as most drying technologies entail application of high temperatures. This causes loss of flavor, nutrients, and bioactive compounds. The color, microstructure, shrinkage, and bulk density of dried fruit products are all affected by drying methods and processing conditions [19, 20].

Drying is a thermal process involving heat and moisture transfer occurring concurrently [21]. Consequently, it is crucial to develop a better understanding of the controlling parameters of the process. Drying process mathematical models are used to design new or improve existing drying systems and monitor the drying process. Although a number of mathematical models have been proposed to explain the drying process, thin-layer drying models are the most widely used [22]. Many researchers have studied and modelled the thin-layer drying of various vegetables and fruits such as mango slices [23], mango puree [24], pears [25], apricot [26], permission fruits [27], and jackfruit [10]. However, to the best of our knowledge, there is no information on mathematical modelling of refractance window drying on jackfruit drying behavior and the dried products' physical characteristics. Therefore, the objectives of this study were to investigate the thin-layer drying characteristics of jackfruit slices and determine the effects of different drying methods (freeze, oven, solar, and RWD) on the functional properties and microstructure of jackfruit powder and slices, respectively.

## 2. Materials and Methods

### 2.1. Sample Preparation and Drying

Mature yellow-fleshed jackfruits (*Artocarpus heterophyllus*) procured from Kayunga district, Uganda, were used for the study. The fruits were stored at room temperature until they ripened. The fruits were then washed using tap water to remove foreign matter from the surface. The cleaned fruits were sectioned, and the arils and seeds separated. The arils were sliced in thin layers of approximately 3-mm thickness [28].

Refractance window drying was done using a hybrid batch scale refractance window dryer (Utility Model reference number UG/U/2020/000012) on a Mylar sheet (k-mac plastics-Type D clear, thickness 0.010 inches). Electricity was used as a source of heat energy to power the drying system. Water temperature was maintained at 93 °C for 62 minutes. Solar drying was conducted in a greenhouse solar dryer for three days (average temperature of 42.7 °C) [7]. Convection oven drying was done in an MRC forced air oven (DFO 150) at 70 °C for 20 hours [29]. Freeze drying was done using a Mini Lyotrap freeze dryer (LTE Scientific Ltd, UK) at 50 Pa with a condenser temperature of − 55°C for 72 hours. Sample mass was recorded periodically during drying at intervals of 30 minutes for oven and solar drying and 5 minutes for refractance window drying [30] using an analytical balance (Uniweigh digital scale) with a precision of ±0.01 g. The experiments were continued until the samples attained constant mass. All experiments were conducted in quadruplicate. Drying kinetics were conducted for three drying methods, solar, oven, and RWD, as the regular sample weighing could not be done during freeze-drying.

### 2.2. Drying Characteristics Analysis

Observed data for the three drying methods are expressed in terms of the moisture ratio [31]:

(1) $$\begin{array}{c} MR=\frac{M-M_{e}}{M_{o}-M_{e}}, \end{array}$$

where *MR* is the moisture ratio, *M* is the moisture content at time *t* (% db), *M*_e_ is the equilibrium moisture content at the condition of the drying air (% db), and M_0_ is the initial moisture content of the sample.

Drying rate at different drying times is determined as the change in moisture content divided by the drying time given by Equation (2) [23]. The drying rate was plotted against drying time (drying rate curve) and against moisture content (Krischer curves) using the observed data for the different drying methods [32].

(2) $$\begin{array}{c} DR=\frac{{MC}_{t+dt}-{MC}_{t}}{dt}, \end{array}$$

where *DR* is the drying rate, *MC*_t_ and *MC*_t+dt_ are the moisture content (dry basis) at time *t* and at *t* + *dt*, respectively (% db.), and *t* is the elapsed drying time (minutes).

A total of fifteen thin-layer drying models were fitted to the observed drying results (Table 1). Model fitting was done by minimizing the total sum of square errors (SSE) between the experimental and the model data. Microsoft Excel 2019 with the solver function and GRG nonlinear solver method was used to calculate and fit the different models to the observed data [33].

Thin-layer drying models were evaluated and compared using the coefficient of determination (*R*^2^) and standard error estimate (SEE) [34, 47]. Higher values of *R*^2^ and lower values of SEE were chosen as the criteria for the goodness of fit (Aregbesola et al., 201; [47, 48]). The values of *R*^2^ and SEE are obtained using Equations (3) and (4), respectively.

(3) $$\begin{array}{c} {\text{R}}^{2}=\frac{\sum_{\text{i}=1}^{\text{N}}{\left({\text{MR}}_{\exp,\text{i}}-{\text{MR}}_{\exp\left(\text{mean}\right),\text{i}}\right)}^{2}-\sum_{\text{i}=1}^{\text{N}}{\left({\text{MR}}_{\text{pre},\text{i}}-{\text{MR}}_{\exp,\text{i}}\right)}^{2}}{\sum_{\text{i}=1}^{\text{N}}{\left({\text{MR}}_{\exp,\text{i}}-{\text{MR}}_{\exp\left(\text{mean}\right),\text{i}}\right)}^{2}}, \end{array}$$

where *MR*_exp,i_ stands for the experimental MR found in any measurement, *MR*_pre,i_ is the predicted MR for this measurement, and *N* is the total number of observations.

(4) $$\begin{array}{c} SEE=\sqrt{\frac{\sum_{i=1}^{N}{\left({MR}_{\exp,i}-{MR}_{pre,i}\right)}^{2}}{N-n_{i}}}, \end{array}$$

where *n*_i_ is the number of constants.

The effective moisture diffusivity (*D*_e_) was determined using Fick's diffusion equation. Since the jackfruit was dried after slicing, the samples were of slab geometry. The effective diffusivity was determined from the expression that relates moisture ratio (MR) and diffusivity given by the following equation [49]:

(5) $$\begin{array}{c} MR=\frac{8}{\pi^{2}}e^{\left(-\frac{\pi^{2}D_{e}t}{4L^{2}}\right)}, \end{array}$$

where *D* is the effective moisture diffusivity (m^2^/s), *L* is the thickness of slice (m), and *t* is the drying time (s).

The effective diffusivity was then calculated from the relationship of the slope (*K*_0_) for the straight line generated on the plotting of logarithmic MR (In MR) against time (*t*) given by the following equation [50]:

(6) $$\begin{array}{c} K_{0}=\frac{\pi^{2}D_{e}}{4L^{2}}. \end{array}$$

### 2.3. Powder Properties

The dried jackfruit flakes were ground into a powder using a Philips Model HR 1727 (Koninklijke Philips N.V., Netherlands) blender and sieved using a stainless steel 600-micron mesh sieve (Endecotts, UK). The milled powder was packaged in resealable bags and stored in airtight containers until further analysis.

#### 2.3.1. Nonenzymatic Browning

Nonenzymatic browning was measured according to the method suggested by Saxena et al. [9]. The extent of browning was evaluated as a nonenzymatic browning index (NEBI). Five (5) grams of jackfruit powder (JFP) sample were extracted with 67% ethanol; the extract was topped up to 100 mL and left to stand for 1 hour at room temperature (24°C ± 2). The extract was filtered through Whatman No. 1 filter paper. NEBI was evaluated spectrophotometrically using a UV–Vis spectrophotometer (Spectroquant Pharo® 300, EU) by measuring absorbance in 10-mm cells against 67% ethanol blank at 420 nm.

#### 2.3.2. Water Solubility Index (WSI)

The WSI of the jackfruit powders (JFP) was determined using the method described by Kha et al. [51] with modifications. Jackfruit powder (2.5 g) and distilled water (30 mL) were vigorously mixed using a vortex mixer (SI-100 N-MRC Lab Equipment, UK) in a 50-mL centrifuge tube for 1 minute, incubated at 37 °C in a water bath (Grant OLS 200,Grant Instruments, UK) for 30 minutes and then centrifuged for 40 minutes at 11,410 g in a Heraeus Megafuge 8 (Thermo Scientific, UK). The supernatant was carefully collected in a preweighed beaker and oven-dried at a temperature of 100 ± 2°C. The WSI (%) was determined by dividing the amount of dried supernatant by the amount of initial 2.5 g jackfruit powder, as shown in the following equation:

(7) $$\begin{array}{c} WSI\;\left(\%\right)=\left(\frac{Dried\;supernatant\;weight}{Initial\;sample\;weight}\right)X100. \end{array}$$

#### 2.3.3. Water Holding Capacity

Water holding capacity was determined using the method proposed by Nguyen et al. [52] with slight modifications. A sample (2.5 g) of JFP was weighed in preweighed 50-mL plastic centrifuge tubes. For each jackfruit powder sample, 10 mL of distilled water was added and well mixed. Samples were left to stand at room temperature (25°C ± 1) for 30 minutes. The mixture was centrifuged at 2852 g for 30 minutes. The supernatant was decanted after centrifugation, and the sample's new mass was registered. WHC (g water/g of powder) is calculated as shown in the following equation:

(8) $$\begin{array}{c} WHC=\frac{Total\;water\;mass}{Dry\;matter\;mass}. \end{array}$$

#### 2.3.4. Oil Holding Capacity

Oil holding capacity was determined using the method proposed by Nguyen et al. [52] with slight modifications. Jackfruit powder (2 g) was weighed in a preweighed 50-mL plastic centrifuge. For each sample, 20 mL of refined vegetable oil (Density 0.955 g/ml) was added and well mixed using a vortex mixer (SI-100 N-MRC Lab Equipment, UK) at the highest speed. The samples were allowed to stand at room temperature for 30 minutes. The sample oil mixture was centrifuged at 2852 g for 30 minutes, the supernatant was carefully decanted, and the new mass of the sample was recorded. Oil holding capacity is calculated as shown in the following equation:

(9) $$\begin{array}{c} OHC=\frac{Mass\;\text{of }sample\;including\;held\;\text{oil}}{Mass\;\text{of }dry\;material}. \end{array}$$

#### 2.3.5. Rehydration Ratio

Rehydration characteristics are affected by processing conditions, sample composition, sample preparation, and the intensity of structural and chemical disruptions caused by drying [53]. Determination of rehydration ratio was based on the method proposed by Shaari et al. [54] with slight modifications. A total of 2.5 g of dried sample was soaked for 60 minutes in 25 mL distilled water, filtered through Whatman filter paper 1, and the filtrates were weighed. The rehydration ratio (R/R) was used to express the fruit powder's ability to absorb water. The rehydration ratio is determined using the following equation:

(10) $$\begin{array}{c} Rehydration\;ratio=\frac{W₂}{W_{1}}, \end{array}$$

where *W*_2_ is the mass of drained material (g) and *W*_1_ is the mass of dried material (g).

#### 2.3.6. Bulk Density

Bulk density (g/mL) was determined by gently adding 2 g of jackfruit powder into an empty 10-mL graduated cylinder. The cylinder was held on a vortex mixer (SI-100 N-MRC Lab Equipment, UK) for 1 minute at the highest speed. The ratio of the mass of the powder and the volume occupied in the cylinder determined the bulk density value [51].

#### 2.3.7. Tapped Density

The tapped density of the samples was measured by placing a 2.5 g powder sample in a 10 mL graduated measuring glass cylinder, which was gently dropped 100 times onto a mat from a height of 15 cm. The tapped density was calculated by dividing the weight of the powder by the tapped volume [51].

#### 2.3.8. True Density

True density was calculated according to Bhusari et al. [55]. Approximately 1 g of jackfruit powder was added to a 10 mL cylinder containing toluene. Then rise in toluene level (mL) was measured, and true density is calculated as

(11) $$\begin{array}{c} True\;density=\frac{Weight\;\text{of }powder\;sample\;\left(g\right)}{Rise\;\text{in }toluene\;volume\;\left(ml\right)}. \end{array}$$

#### 2.3.9. Porosity

The porosity of the powder samples was calculated using the relationship between the bulk and true density of the powder according to Bhusari et al. [55]:

(12) $$\begin{array}{c} Poros\text{i}ty=1-\left(Bulk\;density/True\;density\right). \end{array}$$

#### 2.3.10. Powder Flow Properties

*(1) Hausner Ratio and Carr Index*. The Carr Index and the Hausner Ratio were used to investigate the flow behavior of the JFP sample. The Carr Index and the Hausner Ratio were calculated from the bulk density and tapped density as shown in the following equations [56]:

(13) $$\begin{array}{c} CI=\frac{T_{d}-B_{d}}{T_{d}}X\;100, \end{array}$$

(14) $$\begin{array}{c} HR=\frac{T_{d}}{B_{d}}. \end{array}$$

where *CI* is Carr index, T_d_ is the tapped density, B_d_ is the bulk density, and HR is the Hausner ratio. Different ranges for the Carr index and the Hausner ratio have been defined by Lebrun et al. [57], as presented in Table 2.

### 2.4. Microstructure Analysis

Dried jackfruit slices were secured onto a microscope slide with double-sided adhesive carbon tape and mounted onto the aluminum scanning electron microscope (SEM) holder using more double-sided carbon tape. The samples were sputter-coated. A scanning electron microscope, Zeiss MERLIN (Carl Zeiss Microscopy, Germany), was used at accelerating voltage (EHT) 5 kV (SE2), the working distance of 9.5 mm (SE2), and beam Current of 90 pA (SE2). Fractal dimension (FD) and lacunarity were used to study the structure and irregularities of dried jackfruit. SEM images were analyzed via the FracLac plug-in used in ImageJ software.

### 2.5. Statistical Analysis

All experiments for functional properties were carried out in quadruplicate. Data were subjected to analyses of variance (ANOVA), and multiple comparisons between means were determined using the LSD test (*P* > 0.05) using XLSTAT Version 2020.

## 3. Results and Discussion

### 3.1. Drying Kinetics of Jackfruit Slices

**Figure 1:**
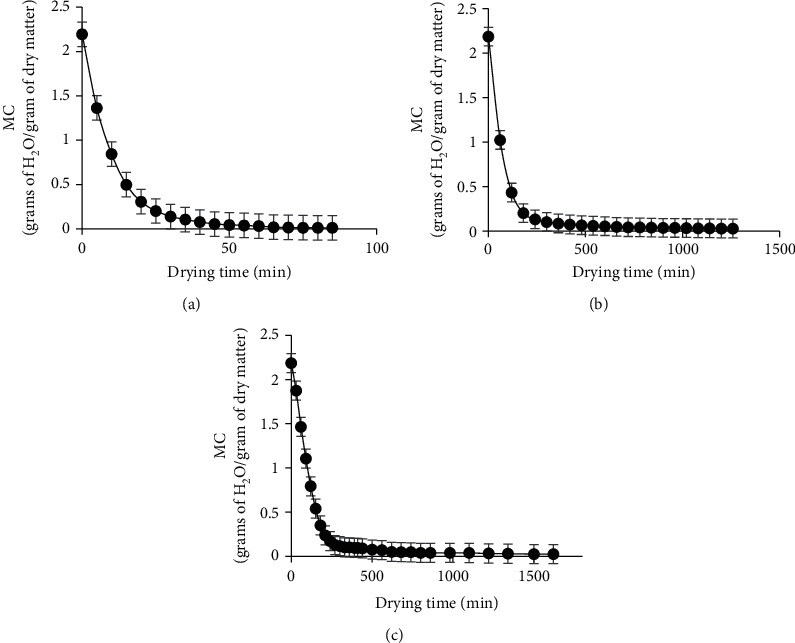
Variation of moisture content with drying time for refractance window drying (a), oven drying (b), and solar drying (c).

Figure 1 shows how the moisture content varies with drying time for RWD, oven drying, and solar drying, respectively. Generally, a nonlinear decrease in moisture content with drying time was recorded for all drying methods. From an average moisture content of 2.185 g/g of dry matter, the samples were reduced to 0.01 g/g of dry matter after 1.42, 21, and 27 hours of effective drying for RWD, oven, and solar drying, respectively.

**Figure 2:**
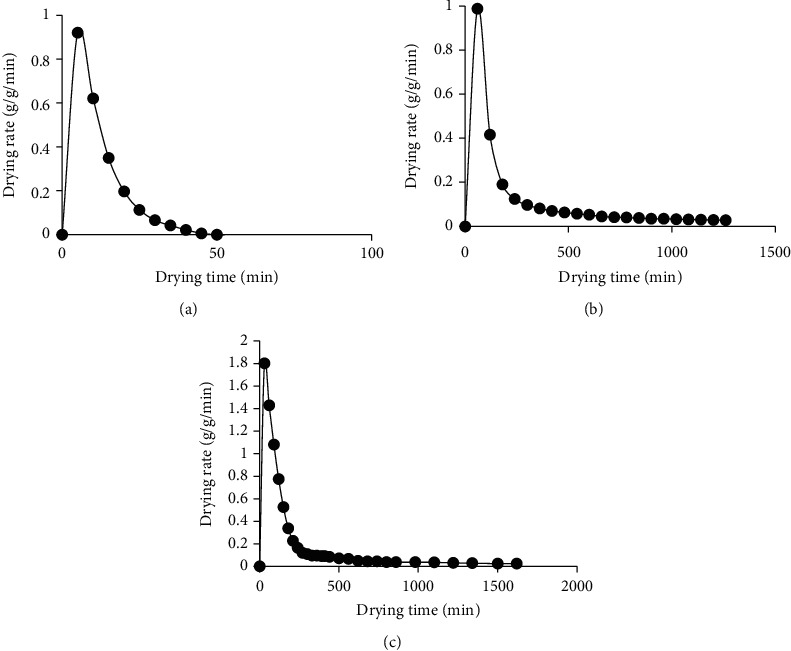
Drying rate curves for refractance window drying (RWD) (a), oven drying (b), and solar drying (c).

**Figure 3:**
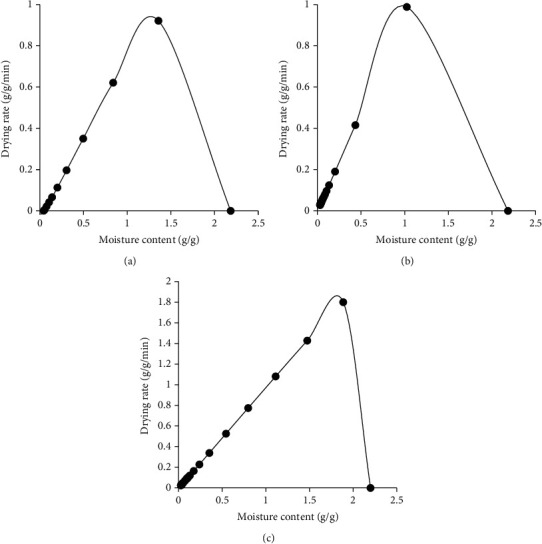
Krischer curves for refractance window drying (a), oven drying (b), and solar drying (c).

The drying rate curves for RWD, oven drying, and solar drying are shown in Figure 2. The figures indicate that the drying rate rapidly increases with time to a maximum value and then decreases. The maximum drying rates are reached after 5, 60, and 30 minutes of drying for RWD, oven, and solar drying, respectively. The rapid drying during refractance window drying could be attributed to the fact that during RW drying, the three modes of heat transfer, conduction, convection, and radiation, are active. Additionally, the maintenance of process water at temperatures just below boiling and thin plastic material with the infrared transmission in the wavelength range that matches the absorption spectrum for water all work together to facilitate rapid drying [58]. This is unlike solar drying, where solar radiation is the main mode of heat transfer. The complexity in solar drying is noteworthy due to changes of climatological factors during the entire drying process, which affect the drying rate. Solar radiation intensity also varies considerably according to the weather conditions and with the hour of the day [59]. The Krischer curves for RWD, oven, and solar drying are shown in Figure 3. The graphs indicate that the drying rate increases steadily from the initial value when the slices are fresh but then increases rapidly to the maximum value and then falls for all drying methods.

### 3.2. Mathematical Modelling of Drying Kinetics

**Figure 4:**
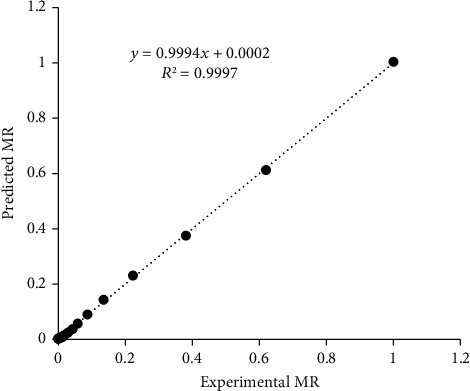
Comparison of predicted moisture ratio (MR) by modified Herndason and Pabis model with observed MR for refractance window drying.

**Figure 5:**
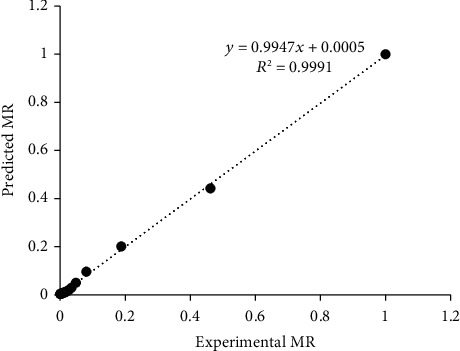
Comparison of predicted moisture ratio (MR) by Verma et al. model with observed MR for oven drying.

**Figure 6:**
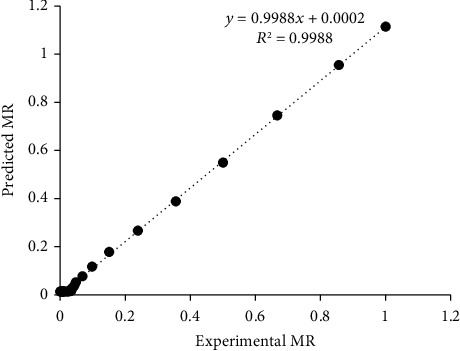
Comparison of predicted moisture ratio (MR) by the Hii et al. model with observed MR for solar drying.

The linear nature of the curve at 45° slope from the origin in the plot of predicted MR against observed MR for Figures 4, 5, and 6 indicates that the models are highly accurate at predicting the drying kinetics of jackfruit for RWD, oven, and solar drying, respectively [60, 61]. The moisture ratio data observed were fitted to the fifteen (15) thin-layer drying models as presented in Table 2. For all models, the *R*^2^ and SEE values ranged between 0.951-0.9997 and 0.0047–0.0606, respectively (Table 3). Most *R*^2^ values were greater than the acceptable *R*^2^ value of 0.97 [62] except for the Haghai and Ghanadzadah model with the oven and solar drying at 0.9618 and 0.9510, respectively. Based on the highest *R*^2^ and lowest SEE values criteria for optimizing the drying models, the models that best fit the observed data were modified Henderson and Pabis, Verma et al., and Hii et al. for RWD, oven, and solar drying, respectively.

**Figure 7:**
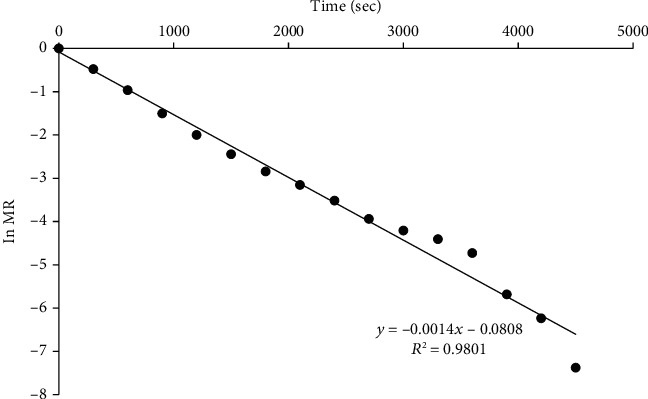
A plot of ln MR and time of jackfruit dried with RWD.

**Figure 8:**
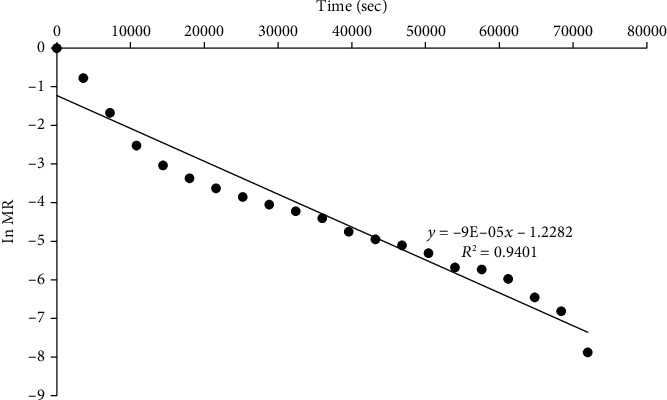
A plot of ln MR and time of jackfruit dried with an oven dryer.

**Figure 9:**
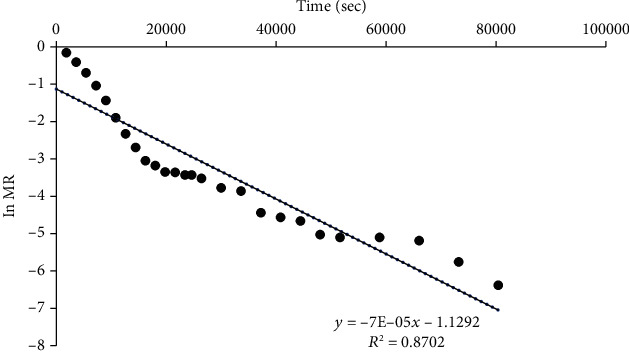
A plot of ln MR and time of jackfruit dried with a solar dryer.

Plots of the logarithm of MR versus time for RWD, oven, and solar dryers are shown in Figures 7, 8, and 9, respectively. Similar results were obtained by Saxena and Dash [63]. From the slopes, effective diffusivity was 5.11 × 10^−9^, 3.28 × 10^−10^, and 2.55 × 10^−10^ m^2^/s for RWD, oven, and solar drying, respectively (Table 4).

### 3.3. Properties of Jackfruit Powders

Researchers were unable to obtain a powder from solar-dried jackfruit. The functional properties of freeze-dried, refractance window dried, and oven-dried powders (Table 5) were evaluated in this study.

#### 3.3.1. Nonenzymatic Browning

Color is influenced by many factors, including fruit variety and ripeness, but particularly by the drying process of the pulp [64]. During pulp dehydration, the product is exposed to high temperatures, which cause enzymatic and nonenzymatic browning (Maillard reactions), which darken the product [65]. In this study, the nonenzymatic browning was highest in oven-dried jackfruit (0.402) and lowest in freeze-dried jackfruit (0.084). A study by Tontul and Topuz [66] reported similar results, with RW drying of pomegranate leather exhibiting a lower browning reaction compared to hot air drying and microwave-assisted hot air drying.

#### 3.3.2. Solubility Index

The water solubility index measures the powder's ability to dissolve in water, where a higher percentage indicates a higher solubility of powder in water [67]. Drying methods significantly affected (*P* > 0.05) the solubility of jackfruit powder. The highest solubility was observed in the freeze-dried JFP (75.7%) and the lowest in the oven-dried JFP (66.1%). The WSI was lower than Wong et al. [68] observed. Laokuldilok and Kanha [69] found that freeze-dried rice powder had better solubility values than spray dried rice samples.

#### 3.3.3. Rehydration Ratio

Rehydration ratio can be used to characterize the destructive degrees of drying conditions on product structure. A smaller degree of structural damage to the dried product results in a better quality of dried product and a higher rehydration ratio [70]. Table 4 shows significant (*P* < 0.05) differences in the rehydration ratios of jackfruit powder obtained from the three drying methods. The freeze-dried jackfruit had the best rehydration capability. The difference in rehydration ability in jackfruit powders could be attributed to the differences in the microstructure. Wang et al. [71] found that the porous structure formed in the drying process of ginger was conducive to the rehydration of the product, and the rehydration ability of the product decreased with an increase in drying temperature. In this study, however, although the process temperatures for RW drying were higher than oven drying, the rehydration capability of RW dried jackfruit powder was higher than that of oven dried jackfruit powder.

#### 3.3.4. Bulk, Tapped and True Density and Porosity

The dehydration process has a significant impact on bulk density. The bulk and tapped densities provide insight into the particle packing and arrangement and the material's compaction profile [72]. The drying process significantly (*P* < 0.05) influenced the bulk density of the jackfruit powder. The bulk density of jackfruit powder ranged from 0.566 g/cm^3^ to 0.699 g/cm^3^, depending on the drying technique. Among all drying techniques, freeze-drying exhibited the highest reduction of the bulk density (Table 4). Mirhosseini and Amid [72], Krokida and Maroulis [73] and Caparino et al. [67] reported similar findings. They determined the bulk densities of freeze-dried apple, banana, potato and carrot materials and mango, respectively, as the lowest. Materials with lower bulk density tend to have higher porosity and vice versa [72]. The freeze-dried jackfruit powder had the lowest bulk density in the current sample, resulting in the highest porosity of all the dried powders. The reduction in the bulk density might significantly affect the solubility of the freeze-dried jackfruit powder. The bulk density of different jackfruit powders was comparable with that reported for pineapple powder (0.579 g/cm^3^) and mango powder (0.638 g/cm^3^) [56].

Powder flow behavior may be inferred from the ratio of bulk and tapped densities [56]. In this study, the tapped density ranged from 0.596/cm^3^ to 0.774/cm^3^, depending on the drying technique. This study revealed that the refractance window dried jackfruit powder had the least tapped density. On the other hand, the oven-dried jackfruit powder had the highest tapped density. The changes in the tapped and true density of the dehydrated products significantly influence powder flow. In this study, the true density varied from 1.671 to 1.895 g/cm^3^. These values were higher than the true density reported for pineapple powder (1.35 g/cm^3^) and mango powder (1.36 g/cm^3^) [56]. In this study, RWD dried jackfruit powder exhibited the lowest tapped density.

#### 3.3.5. Powder Flow Properties of Fruit Powders

A powdered material's flowability, as determined by the Carr index and Hausner ratio, is a significant characteristic. The physical properties of the powder, such as particle size and shape, surface structure, particle density, and bulk density, all influence flowability [56]. According to Tze et al. [74], flowability greatly influences transportation, formulation and mixing, compression, and packaging. Oven-dried and RW dried jackfruit powders exhibited excellent flowability, while freeze-dried powders exhibited good flowability. This could be due to the small mean particle size demonstrated by the high Hausner ratio and Carr index. Tze et al. [74] concluded that powder with smaller particle size has poor flowing properties.

#### 3.3.6. Water Holding Capacity and Oil Holding Capacity

Water holding capacity (WHC) and oil holding capacity (OHC) are technological parameters that give insight into the potential to incorporate jackfruit powder in other food matrices. Freeze fried jackfruit powder had a WHC of 2.011 g/g followed by oven-dried powder (1.445 g/g) and refractance window dried jackfruit powder (1.238 g/g). There was no difference between oven and refractance window dried jackfruit powders. A similar trend was observed for OHC, which was highest in freeze-dried jackfruit powder (1.137 g/g) and lowest in RWD powder (0.827 g/g). Incorporating powder with a high WHC can improve the technological characteristics of the food products, such as decreasing the calories and syneresis while changing the viscosity and texture of the final product [75]. The OHC of the powder depends on the chemical and physical structures of the polysaccharides. This property is important to avoid fat loss during the cooking process; consequently, it has an auxiliary use in flavor preservation. According to Selani et al. [76], ingredients with a high OHC cause high-fat food products and emulsions to be stabilized. Jackfruit powder does not have the ability to be used as an ingredient for these purposes due to its low OHC values.

### 3.4. Microstructure

**Figure 10:**
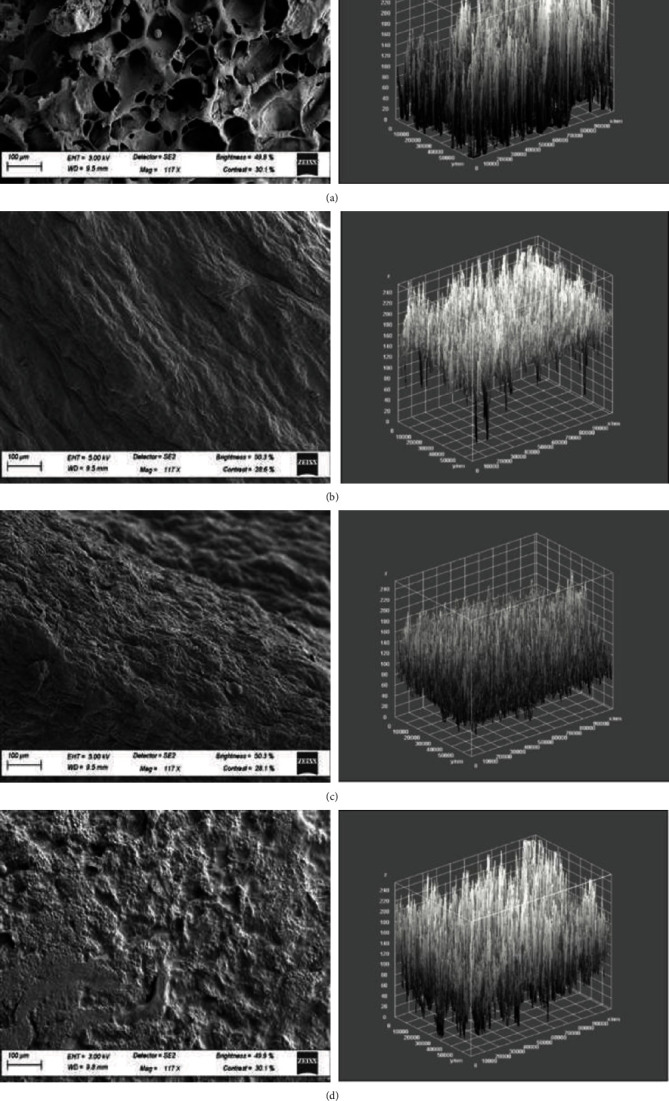
Cross-section microstructure (117 × mag) of jackfruit dried using different drying methods and their respective grey-level intensity plots: (a) Freeze-dried (FD); (b) oven-dried (OD); (c) refractance window dried (RWD); (d) solar dried (SD).

The microstructures of the jackfruit slices obtained by scanning electron microscopy are shown in Figure 10. Porous structures were observed in the freeze-dried samples compared to the other drying methods. This occurs because the ice in the material helps prevent shrinkage and collapse of the structure and shape during freeze-drying, resulting in minor volume changes [67]. Conspicuous changes in the shape and size of cells were observed in the oven, solar, and RWD jackfruit slices. The dehydration temperature and rate greatly influence the texture of the food and, in general, faster processes and higher temperatures cause more significant changes. At a high drying rate, the damage to tissue structure is much greater, and, as a result, the material becomes fragile. Tissue damage creates more significant shrinkage stress when compared to that at low drying rates [20]. In this study, the maximum drying rates were reached after 5, 60, and 30 minutes of drying for RWD, oven, and solar drying, respectively. The rapid drying rate achieved by RWD could be attributed to the fact that during RW drying, the three modes of heat transfer, conduction, convection, and radiation, are active. Owing to the lack of liquid water and the low temperature used, freeze-drying is thought to protect the primary structure and prevent shrinkage [77]. Consequently, a porous structure with little or no shrinkage, which can rehydrate readily before use, is obtained [19].

Fractal dimension ranged from 1.837 in solar dried jackfruit to 1.809 in freeze-dried jackfruit. Lacunarity ranged from 0.258 in solar dried jackfruit and 0.404 in freeze-dried jackfruit (Table 6). The parameters analyzed were intercorrelated and demonstrated a high degree of correlation with porosity (0.875), which plays an important role in texture perception [78], so it can be assumed that the variables defined and the method described in this work can be regarded as good tools for future study of the relationship between microstructure and the final product texture. Additionally, the high correlation coefficients, especially those related to porosity, confirm the capacity of scanning electron microscopy and image analysis to predict final dried jackfruit characteristics if equations relating to microstructure and functional parameters are developed [79].

## 4. Conclusions

The drying kinetics and effects of different drying methods (SD, OD, FD, and RWD) on functional properties and microstructure of jackfruit were examined experimentally. The maximum drying rates were reached after 5, 60, and 30 minutes for RWD, oven, and solar drying, respectively. The models that best fit the observed data were Modified Henderson and Pabis, Verma et al., and Hii et al. for RWD, oven, and solar drying, respectively. The results showed that RWD is a promising drying method for jackfruit quality preservation, as it allowed for a less nonenzymatic browning than OD and SD. RWD powder had a better rehydration ratio than OD but was lower than FD. The results suggest that the RWD jackfruit had relatively better quality in terms of functional properties than SD and OD, comparable to FD, and is a faster drying method than SD, OD, and FD. Therefore, RWD is an alternative for the production of high-quality dried jackfruit.

## Figures and Tables

**Table 1:** Thin layer drying models used in the study.

S/N	Model name	Model	Reference
1	Newton	MR = exp (−kt)	Aregbesola et al. [34]
2	Page	MR = exp (−kt^n^)	Akoy [23]
3	Modified page	MR = exp (−kt)^n^	Sobukola and Dairu [35]
4	Henderson and Pabis	MR = a exp (−kt)	Meisami-Asl et al. [36]
5	Modified Henderson and Pabis	MR = a exp (−kt) + b exp (−gt) + c exp (−ht)	Taheri-Garavand et al. [37]
6	Silva et al.	MR = exp(−at − b√t)	Da Silva et al. [38]
7	Logarithmic	MR = a exp (−kt) + b	Inyang et al. [39]
8	Two term	MR = a exp (−k_0_t) + b exp (−k_1_t)	Afolabi et al. [40]
9	Two term exponential	MR = a exp (−kt) + (1 − a) exp (−kat)	Mezquita, López, and Muñoz [41]
10	Verma et al.	MR = a exp (−kt) + (1 − a) exp (−gt)	Akinola and Ezeorah [42]
11	Diffusion approach	MR = a exp (−kt) + (1 − a) exp (−kbt)	Sobukola et al. [43]
12	Midilli et al.	MR = a exp (−kt^n^) + bt	Iwe et al. [44]
13	Modified Midilli et al.	MR = a exp (−kt) + b	Onwude et al. [22]
14	Hii et al.	MR = a exp (−k_1_t^n^) + b exp (−k_2_t^n^)	Kumar et al. [45]
15	Haghai and Ghanadzadeh	MR = a exp (−bt^c^) + dt^2^ + et + f	Haghi and Ghanadzadeh [46]

**Table 2:** Flowability classification.

Flowability	Carr index (CI), %	Hausner ratio (HR)
Excellent	0–10	1.00–1.11
Good	11–15	1.12–1.18
Fair	16–20	1.19–1.25
Passable	21–25	1.26–1.34
Poor	26–31	1.35–1.45
Very poor	32–37	1.46–1.59
Very, very poor	>38	>1.60

**Table 3:** Model coefficients and the goodness of fit for the different drying methods.

S/N	Model	Drying method	Parameters	*R* ^2^	SEE
1	Newton	RWD	*k* = 0.0972	0.9996	0.0054
		Oven	*k* = 0.0133	0.9987	0.0086
		Solar	*k* = 0.0089	0.9879	0.0301
2	Page	RWD	*K* = 0.0981, *n* = 0.9964	0.9996	0.0054
		Oven	*K* = 0.0129, *n* = 1.0063	0.9987	0.0085
		Solar	*K* = 0.0017, *n* = 1.3455	0.9984	0.0110
3	Modified page	RWD	*K* = 0.1394, *n* = 6972	0.9996	0.0054
		Oven	*K* = 0.0516, *n* = 0.2578	0.9987	0.0085
		Solar	*K* = 0.0134, *n* = 0.6683	0.9879	0.0301
4	Henderson and Pabis	RWD	*K* = 0.0973, *a* = 1.0011	0.9996	0.0053
		Oven	*K* = 0.0133, *a* = 1.0016	0.9987	0.0085
		Solar	*K* = 0.0095, *a* = 1.0694	0.9888	0.0290
5	Modified Henderson and Pabis	RWD	*K* = 0.0243, *a* = 0.0194, *b* = 0, *c* = 0.9843, *g* = 0.641, *h* = 0.1006	0.9997	**0.0047**
		Oven	*K* = 0.0133, *a* = 1.0015, *b* = 0, *c* = 0, *g* = 1.0002, *h* = 1.0001	0.9987	0.0085
		Solar	*K* = 0.0095, *a* = 1.0694, *b* = 0, *c* = 0, *g* = 0.6410, *h* = 0.0726	0.9888	0.0290
6	Silva et al.	RWD	*a* = 0.0972, *b* = 0	0.9996	0.0054
		Oven	*a* = 0.0133, *b* = 0	0.9987	0.0086
		Solar	*a* = 0.0089, *b* = 0	0.9879	0.0301
7	Logarithm	RWD	*K* = 0.0987, *a* = 0.9985, *c* = 0.0041	0.9997	0.0050
		Oven	*K* = 0.0136, *a* = 0.9958, *c* = 0.0073	0.9988	0.0082
		Solar	*K* = 0.0095, *a* = 1.0694, *c* = 0	0.9888	0.0290
8	Two term	RWD	*a* = 0.7399, *b* = 0.2633, k_0_ = 0.0896, k_1_ = 0.1260	0.9996	0.0054
		Oven	*a* = 1.0016, *b* = 0, k_0_ = 0.0133, k_1_ = 0.1179	0.9987	0.0085
		Solar	*a* = 1.0694, *b* = 0, k_0_ = 0.0095, k_1_ = 0.0934	0.9888	0.0290
9	Two term exponential	RWD	*K* = 1, *a* = 0.97	0.9996	0.0053
		Oven	*K* = 0.0133, *a* = 0.9731	0.9987	0.0086
		Solar	*K* = 0.0133, *a* = 0.9237	0.9985	0.0105
10	Verma et al.	RWD	*K* = 0.0225, *a* = 0.0167, *g* = 0.0999	0.9997	0.0048
		Oven	*K* = 0.014, *a* = 0.9751, *g* = 0.0017	**0.9991**	**0.0071**
		Solar	*K* = 0.0129, *a* = 1.7738, *g* = 0.0275	0.9985	0.0107
11	Diffusion approach	RWD	*K* = 0.1228, *a* = 0.0981, *b* = 0.7733	0.9996	0.0054
		Oven	*K* = 0.0133, *a* = 2.00, *b* = 0.9999	0.9987	0.0086
		Solar	*K* = 0.0089, *a* = 0.0279, *b* = 1.0000	0.9879	0.0301
12	Midilli et al.	RWD	*K* = 0.0967, *n* = 1.0045, *a* = 1.0012, *b* = 0.00005	0.9996	0.0053
		Oven	*K* = 0.0123, *n* = 1.0179, *a* = 1.0012, *b* = 0.000006	0.9985	0.0091
		Solar	*K* = 0.0016, *n* = 1.3495, *a* = 1.0036, *b* = 0.000008	0.9981	0.0119
13	Modified Midilli et al.	RWD	*K* = 0.0987, *a* = 0.9985, *b* = 0.0041	0.9997	0.0050
		Oven	*K* = 0.0109, *a* = 0, *b* = 0.0079	0.9967	0.0135
		Solar	*K* = 0.0095, *a* = 1.0694, *b* = 0	0.9988	0.0290
14	Hii et al.	RWD	*n* = 0.9948, *a* = 0.5009, *b* = 0.5009, k_1_ = 0.0987, k_2_ = 0.0987	0.9996	0.0054
		Oven	*n* = 1.0052, *a* = 0.5007, *b* = 0.5007, k_1_ = 0.0129, k_2_ = 0.0129	0.9987	0.0084
		Solar	*n* = 1.4087, *a* = 0.0176, *b* = 0.9823, k_1_ = 0.2901, k_2_ = 0.0013	**0.9988**	**0.0094**
15	Haghai and Ghanadzadeh	RWD	*a* = 0.9428, *b* = 0.0433, *c* = 1.2944, *d* = 0.0000005, *e* = 0.00001, *f* = 0.0035	0.9930	0.0232
		Oven	*a* = 0.9428, *b* = 0.0433, *c* = 1.2944, *d* = 0.0000005, *e* = 0.00001, *f* = 0.0035	0.9618	0.0456
		Solar	*a* = 1.005, *b* = 0.0388, *c* = 0.7, *d* = 0, *e* = 0, *f* = 0.00000009	0.9510	0.0606

**Table 4:** Diffusivities of jackfruit slices with different drying methods.

Drying method	Diffusivity (m^2^/s)	*R* ^2^
RWD	5.11 × 10^−9^	0.9801
Oven dryer	3.28 × 10^−10^	0.9401
Solar dryer	2.55 × 10^−10^	0.8702

**Table 5:** Properties of jackfruit powders obtained using different drying methods.

Parameter	Freeze-dried	Oven-dried	RWD
Moisture content	2.599 ± 0.03^a^	2.261 ± 0.11^a^	3.776 ± 0.09^a^
Solubility (%)	75.70 ± 1.7^b^	66.06 ± 1.15^a^	73.22 ± 1.04^b^
Nonenzymatic browning (420 nm)	0.084 ± 0.00^b^	0.402 ± 0.01^d^	0.133 ± 0.01^c^
True density (g/cm^3^)	1.674 ± 0.01^a^	1.895 ± 0.19^a^	1.671 ± 0.01^a^
Water holding capacity (g/g)	2.011 ± 0.08^b^	1.445 ± 0.05^a^	1.238 ± 0.10^a^
Oil holding capacity (g/g)	1.137 ± 0.14^a^	0.946 ± 0.09^a^	0.827 ± 0.01^a^
Bulk density (g/cm^3^)	0.566 ± 0.01^a^	0.699 ± 0.03^c^	0.591 ± 0.00^b^
Rehydration ratio	5.791 ± 0.70^b^	1.954 ± 0.24^a^	4.182 ± 0.77^ab^
Porosity	0.662^c^	0.631^a^	0.646^b^
Tapped density (g/cm^3^)	0.650 ± 0.01^b^	0.774 ± 0.01^c^	0.596 ± 0.00^a^
Carr index	12.82	9.63	0.87
Hausner ratio	1.15	1.11	1.01
Flowability	Good	Excellent	Excellent

**Table 6:** Fractal dimension and lacunarity of jackfruit dried with different methods.

Drying methods	Fractal dimension	Lacunarity
Freeze dried	1.809 ± 0.03	0.404 ± 0.05
Oven dried	1.836 ± 0.03	0.290 ± 0.04
Refractance window dried	1.812 ± 0.03	0.395 ± 0.04
Solar dried	1.837 ± 0.03	0.258 ± 0.03

## Data Availability

The data used to support the findings of this study are available from the corresponding author upon request.
